# Digital Pathology Tailored for Assessment of Liver Biopsies

**DOI:** 10.3390/biomedicines13040846

**Published:** 2025-04-01

**Authors:** Alina-Iuliana Onoiu, David Parada Domínguez, Jorge Joven

**Affiliations:** 1 Unitat de Recerca Biomèdica, Hospital Universitari Sant Joan, Universitat Rovira i Virgili, 43204 Reus, Spain; david.parada@urv.cat; 2 Department of Medicine and Surgery, Faculty of Medicine, Universitat Rovira i Virgili, 43201 Reus, Spain; 3 Department of Pathology, Hospital Universitari Sant Joan, 43204 Reus, Spain; 4 The Campus of International Excellence Southern Catalonia, 43003 Tarragona, Spain

**Keywords:** algorithms, deep learning, liver, obesity, virtual images, whole tissue slide

## Abstract

Improved image quality, better scanners, innovative software technologies, enhanced computational power, superior network connectivity, and the ease of virtual image reproduction and distribution are driving the potential use of digital pathology for diagnosis and education. Although relatively common in clinical oncology, its application in liver pathology is under development. Digital pathology and improving subjective histologic scoring systems could be essential in managing obesity-associated steatotic liver disease. The increasing use of digital pathology in analyzing liver specimens is particularly intriguing as it may offer a more detailed view of liver biology and eliminate the incomplete measurement of treatment responses in clinical trials. The objective and automated quantification of histological results may help establish standardized diagnosis, treatment, and assessment protocols, providing a foundation for personalized patient care. Our experience with artificial intelligence (AI)-based software enhances reproducibility and accuracy, enabling continuous scoring and detecting subtle changes that indicate disease progression or regression. Ongoing validation highlights the need for collaboration between pathologists and AI developers. Concurrently, automated image analysis can address issues related to the historical failure of clinical trials stemming from challenges in histologic assessment. We discuss how these novel tools can be incorporated into liver research and complement post-diagnosis scenarios where quantification is necessary, thus clarifying the evolving role of digital pathology in the field.

## 1. Introduction

### 1.1. Obesity and Associated Comorbidities: A Public Health Priority

Since 1990, the global adult obesity rate has more than doubled, while adolescent obesity has quadrupled. By 2022, approximately 2.5 billion adults were classified as overweight, with 890 million living with obesity. Metabolic dysfunction-associated steatotic liver disease (MASLD) affects up to 25% of the global population and is closely tied to obesity. These interconnected, largely preventable pandemics account for over 15% of healthcare system expenditures in many Western countries [1,2].

### 1.2. Expanding Concepts in MASLD

The terminology surrounding liver diseases has evolved recently, with new terms introduced in 2023. Previously, these conditions were known as Non-Alcoholic Fatty Liver Disease (NAFLD) and Non-Alcoholic Steatohepatitis (NASH). The shift to terms like MASLD and MASH aims to more accurately describe the metabolic dysfunctions that lead to these liver diseases. In obesity, there is a total overlap between the populations classified as MASLD and those previously identified as NAFLD. As the disease progresses, patients may develop Metabolic Dysfunction-Associated Steatohepatitis (MASH), a more severe form characterized by liver inflammation, hepatocyte ballooning, and fibrosis [3,4,5]. According to the multi-society Delphi consensus statement, MASLD refers to individuals with hepatic steatosis and at least one cardiometabolic risk factor. These factors include the following: (1) Body Mass Index (BMI) of 25 kg/m^2^ or higher or a waist circumference exceeding 94 cm for males and 80 cm for females, adjusted by ethnicity; (2) fasting serum glucose levels of 5.6 mmol/L or higher, 2 h post-load glucose levels of 7.8 mmol/L or higher, HbA1c of 5.7% or higher, a diagnosis of type 2 diabetes, or ongoing treatment for type 2 diabetes; (3) blood pressure of 130/85 mmHg or higher or specific antihypertensive medications; (4) plasma triglycerides of 1.70 mmol/L or higher, or treatment with lipid-lowering medications; (5) plasma HDL-cholesterol levels of 1.0 mmol/L or lower for males and 1.3 mmol/L or lower for females, or lipid-lowering treatment.

### 1.3. Histological Assessment of Liver Biopsies Is a Significant Drawback to Finding a Cure for MASH

In 1980, a group of pathologists made a significant breakthrough by describing findings in 20 patients with previously unnamed liver disease of unknown cause. The biopsies revealed fatty changes with mixed inflammatory infiltrates and evidence of fibrosis [6]. Their findings led to the initial classification of non-alcoholic fatty liver (NAFL) and NASH, a milestone in the understanding of liver diseases. This initial dual classification soon became inadequate as the lesions covered a heterogeneous group of chronic and progressive liver conditions in different combinations and varying severities [7]. The risk of disease progression correlates with the fibrosis stage and the presence of MASH [8]. Finding a cure for MASH necessitates clinical trials and liver biopsies. These procedures remain the gold standard but are costly and tedious, with potential sampling errors and risk of complications. Pathologists traditionally use several scoring systems to address these complexities using the unweighted sum of semiquantitative, primarily subjective, measures of steatosis, hepatocellular ballooning, fibrosis, and lobular inflammation [9,10,11]. However, clinical trials require accurate and precise quantification of specific features, and evidence indicates that even among pathologists with high expertise, intraobserver and interobserver agreement in quantifying the aforementioned histologic features is modest [12]. The limitations in these methods underscore the urgent and crucial need for improved, computer-aided quantification in liver disease diagnostics. Segmentation procedures must also be improved. Developments in this field include the study of other diseases [13,14].

## 2. Advantage of Computational Histology for Assessing Outcomes

Despite their limitations, conventional histopathological evaluations are still conditionally used as surrogate endpoints for trial enrollment, stratification, and assessment. So far, only one drug, Resmetirom, has been approved for specific indications in the management of MASH [15]. However, the limited sensitivity of scoring systems and the variability in manual assessments of histology-based endpoints have resulted in an incomplete evaluation of treatment responses, contributing to clinical trial failures and challenges in identifying suitable study populations [16,17]. Computer-aided image analysis can minimize discrepancies in quantifying differences. It is essential to compare results from patient recruitment to those at the end of the study in clinical trials to ensure an accurate assessment of relevant features. In liver pathology, advances in digitizing tissue slides and AI progress pave the way for integrating computational pathology into clinical practice. Specifically, fibrosis assessment to standardize collagen distribution is essential, and ongoing research focuses on digital pathology and the combination of second harmonic generation microscopy with two-photon excited fluorescence [18,19]. Computational histology can help circumvent limitations in finding appropriate management strategies for MASH management, and emerging scientific techniques, such as single-cell RNA sequencing and spatial transcriptomics, increase the likelihood of obtaining significant insights into MASH pathophysiology [20,21].

While the implementation of digital pathology in clinical practice is ongoing, the existence of several commercially available scanners makes it a technology of the present [22]. These scanners, optical microscopes, and digital cameras connected to a computer with software that creates virtual images have varying capabilities, especially in image resolution. The process yields high-resolution images ideal for applying algorithms, enabling time-efficient, sensitive, more easily shared and annotated, and specific histopathologic assessments [23]. The role of digital imaging techniques, including new algorithms, in quantifying histopathological aspects of cell changes in hepatocellular carcinoma and liver transplantation medicine is significant and promising [24,25].

Although digital pathology holds great promise, several challenges must be addressed for its widespread adoption. One of the main obstacles is the significant investment required in IT infrastructure, which can be a major issue for resource-limited systems. This includes the need for secure data storage systems and high-speed networks capable of handling the large file sizes associated with digital slides. Standardizing image formats and data protocols is critical to ensure interoperability across different systems and institutions. Regulatory approval for using digital pathology in primary diagnosis is also still pending in many countries, which can delay its implementation.

Furthermore, the use of digital pathology and AI tools raises ethical concerns, particularly regarding patient transparency regarding the accuracy, limitations, and privacy measures of these technologies. This poses a challenge to informed decision-making and trust in the physician–patient relationship.

While these challenges are considerable, the potential benefits of digital pathology underscore the importance of addressing these issues for its successful integration into clinical practice [26,27,28].

## 3. Improvements in Artificial Intelligence May Be the Next Step Toward Precision Pathology

**Figure 1:**
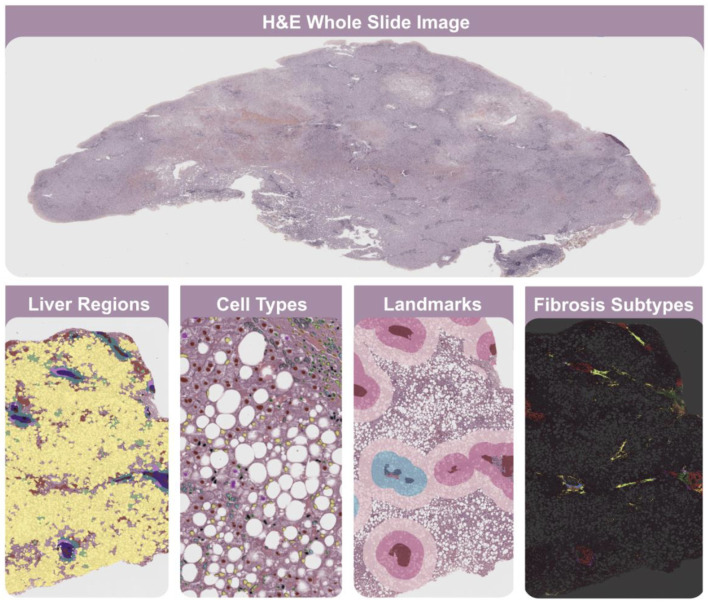
Liver Explore. This AI-powered software provides >1000 quantitative human-interpretable features that characterize liver tissue biopsy microarchitecture from H&E whole slide images. It enables the exploration of cell types in selected liver regions, including immune cells and normal, ballooned, and steatotic hepatocytes. It facilitates the detection of bile ducts, blood vessels, portal tracts, and delimitation of zones 1–3. It is also important to highlight that it consistently reveals portal, periportal, perisinusoidal, and nodular fibrosis, facilitating the assessment of fibrosis subtyping.

Artificial intelligence techniques can potentially revolutionize histopathological evaluations in evaluating algorithms for feature recognition, most notably for counting, providing new opportunities for accurate and efficient assessments in MASH. In our hands, challenges associated with assessing changes in obesity–MASH interactions after weight loss are greatly improved through the image management system of digital pathology labs from AISight™ [29]. In particular, Liver Explore™ is an AI-powered algorithm designed to analyze H&E-stained liver tissue slides (Figure 1). This novel tool remains for research use only, not for use in diagnostic procedures, and consequently has not yet received regulatory approval [30]. However, available data suggest a significant role in resolving the precision and accuracy gaps in MASH assessment [31,32].

Several steps deploy the model: Digitized images annotated by pathologists identify histologic features, excluding artifacts usually caused by defective staining, and image segmentation generates unbiased predictions for each feature. Combining convolutional neural network and graph neural network models generates different categories of histologic readouts. Retrospective studies demonstrate the higher performance of AI-based tools like AIM-MASH™ over expert MASH pathologists. AIM-MASH™, recently qualified by the European Medicines Agency, demonstrated high repeatability and reproducibility across several key assessments in MASH evaluation. AIM-MASH has a 97% agreement with consensus for F4 versus F1-F3 fibrosis stages, compared to 96% for pathologists. Similarly, for NASH resolution without fibrosis worsening, AIM-MASH achieves 86% agreement, while pathologists reach 82%. The tool also excels in fibrosis improvement without NASH worsening, with an 80% agreement, matching pathologists’ performance. Moreover, there is consensus on the superiority of AI-based automation on both enrollment criteria and endpoint assessment, which is important in clinical trials [33,34,35]. These tools are also important in establishing the actual value of non-invasive liver tests and the need to consider multiple patient characteristics in liver diagnosis [36].

## 4. A Guide to Inform the Relevant Histologic Features Associated with the Assessment of MASH: Potential Areas of Research

Correct MASH diagnosis requires measurement of four histologic features: macrovesicular steatosis, hepatocellular ballooning, lobular inflammation, and fibrosis. Each has its respective challenges [27].

### 4.1. Hepatic Steatosis Evaluation

**Figure 2:**
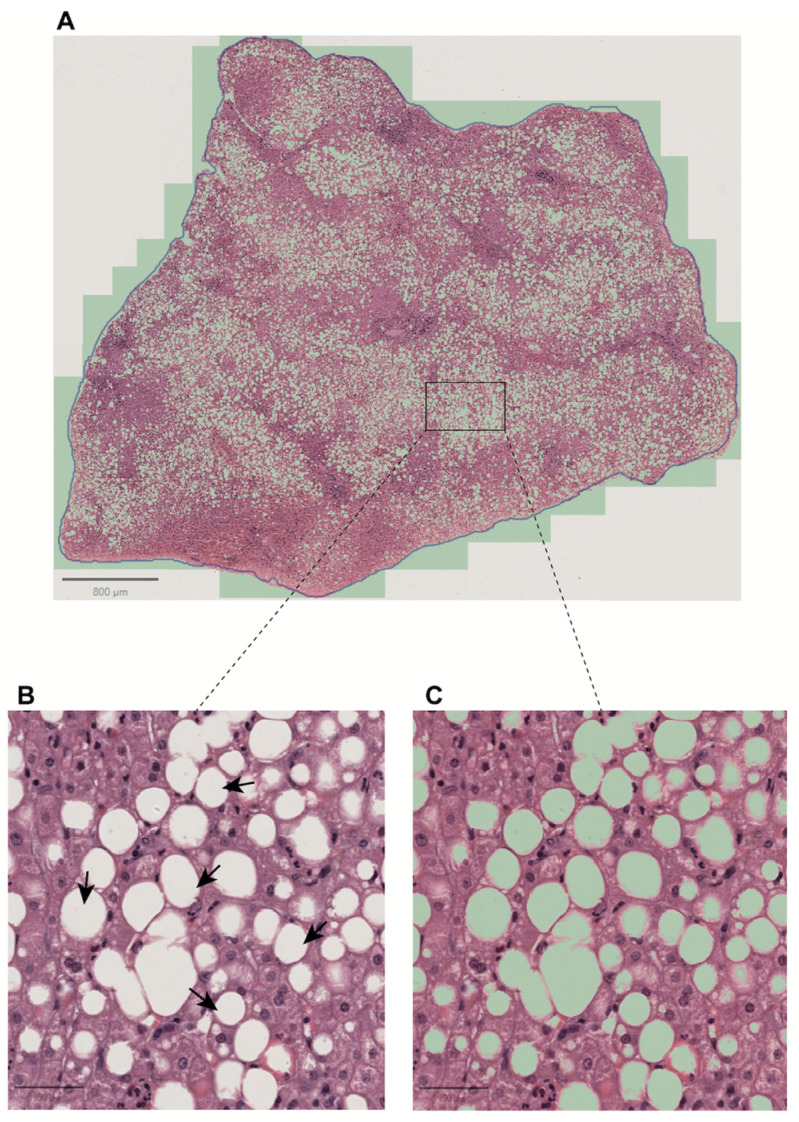
Steatosis assessment. Traditionally, hepatic steatosis evaluates fat buildup. However, there are numerous other critical parameters specific to each lipid droplet, such as the size of each droplet, its spatial distribution, and the density of lipid droplets. Integrating digital pathology with artificial intelligence allows for the extraction and comprehensive evaluation of these features, providing a more detailed and accurate assessment of fatty liver disease. (**A**) Whole tissue slide from a liver biopsy with the steatosis detection threshold using deep learning in QuPath applied in green. (**B**) 20× magnified portion of the tissue slide, showing areas of steatosis indicated by arrows. (**C**) This image is the same as in (**B**), with steatosis areas highlighted in green. It is important to note that an object classifier must be applied to filter out incorrect detections, a crucial step in the process.

Liver biopsy is currently the most reliable method for determining the distribution and amount of fat in the liver; non-invasive diagnostic methods may complement this procedure. These methods can provide similar or even more accurate results than liver biopsy without the associated risks and discomfort. The condition worsens when fat or lipid droplets accumulate in more than 5% of liver cells and concentrate in the centrilobular region (zone 3). In macrovesicular steatosis, large lipid droplets push the cell nucleus to the edge, while in microvesicular steatosis, the cytoplasm becomes foamy, and the nucleus stays in the center. Mixed steatosis, a combination of macrovesicular and microvesicular steatosis, is often involved in MASH. As cirrhosis progresses, steatosis levels decrease, and MASH may be underdiagnosed in advanced liver disease. MASH is the underlying cause of 40% to 80% of cases of cryptogenic cirrhosis. Therefore, underdiagnosis should be avoided to prevent delayed or inadequate treatment. Conversely, although this is probably irrelevant, we consistently find disagreements in human pathology associated with defective assessment of steatotic areas. In this area, open-source software tools like Qupath™ simplify steatosis detection. These tools can extract whole tissue components, identify and separate overlapped steatosis components, and differentiate similar objects to prevent misclassification. It is important to develop new grading systems to assess steatosis accurately, including zonated quantification and the identification of differential steatosis patterns (Figure 2).

### 4.2. Ballooned Hepatocyte Feature Recognition

**Figure 3:**
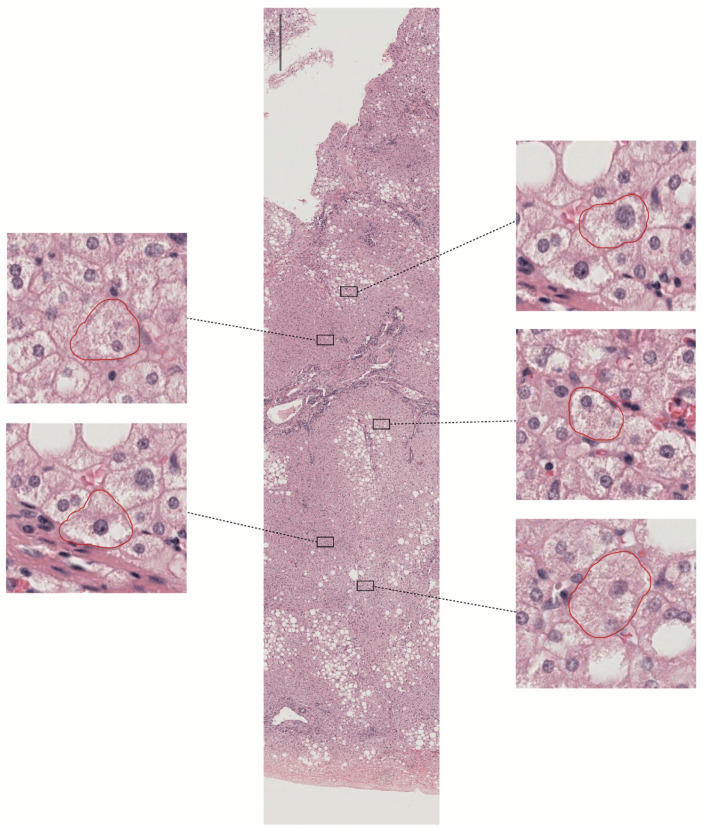
Ballooning is a unique form of hepatocyte injury. Due to its diffuse nature and subjective human detection, it is difficult to detect using deep learning tools. The image in the center shows a whole tissue slide from a liver biopsy at 3× magnification. Annotation tools highlight five potential ballooned hepatocytes for review in the lateral sections (at 40× magnification).

Ballooning refers to large round cells with characteristic reticulated cytoplasm (Figure 3). There remains substantial divergence among hepatopathologists regarding which cells constitute ballooned hepatocytes, suggesting that ballooning represents a morphology spectrum. Indeed, their presence or absence is difficult to assess and an essential feature in assessing treatment outcomes. However, the NASH Clinical Research Network does not explicitly define ballooning, and the guidance for scoring severity uses the generic descriptors ‘few’ or ‘many.’ These restrictions make it challenging to define MASH resolution in clinical trials.

The main feature of ballooning is a high degree of cellular swelling. In MASH, the damaged hepatocytes continuously transition from mild edema to ballooning, lysis, and necrosis. In clinical practice, expert liver pathologists recognize multiple visual cues when assessing the presence of ballooning, which coexists with lipid droplets. Ballooning is usually noted first in zone 3, near the central vein. There may be lobular inflammation and perisinusoidal fibrosis in the immediate vicinity, and ballooned hepatocytes accumulate in areas of matrix deposition. The nature of ballooning as an adaptive or degenerative change is still debatable. However, it is undoubted that ballooning is the most challenging histologic feature regarding interobserver variability.

Given the real challenges of studying ballooning, there is an expectation for breakthroughs in training algorithms through AI/ML-based approaches. At this stage in the research, the limitations and usefulness of an ongoing concordance atlas are significant [23,37]. The goal is to achieve an entirely error-free classification, which is the only method to improve the development of more precise treatments for MASH. Until then, and since not all treatment options work for everyone, the potential benefits of combining therapies that influence weight loss and have independent metabolic effects are encouraging. This approach may be more beneficial and should motivate further research and exploration.

### 4.3. Assessment of Inflammatory Activity

The infiltration of inflammatory cells in liver lobules is an important histologic feature in MASH. The NASH Clinical Research Network uses subjective and semiquantitative scoring. The degree of lobular inflammation is scored from 0 to 3 based on the number of foci showing lobular inflammation per 20× fields. A focus of lobular inflammation is defined as two or more inflammatory cells within the sinusoids or surrounding injured hepatocytes. However, variability may be amplified for cases near a grading cutoff and may worsen based on the observer’s skills. Similarly, semiquantitative grades may fail to show improvement after treatment accurately. Therefore, exploring how digital pathology and straightforward algorithms can incorporate automated, quantitative, and morphometric measurements of inflammatory burden may be helpful. 

Since inflammatory cells are smaller than the surrounding cells, the number of nuclei in inflamed areas is higher. Consequently, a higher concentration of these cells leads to increased nuclear density, which correlates strongly with grading scores and has the potential to enhance current assessment methods [38]. Displaying images stained with different techniques is often advantageous. Incorporating immunohistochemistry (IHC) markers such as CD15, CD68, CD4, and CD8 offers valuable insights into the immune responses in MASH. For instance, CD15, typically linked to neutrophils, helps identify acute inflammatory responses, while CD68 marks macrophages, indicating chronic inflammation and fibrosis. CD4 and CD8, as markers for helper and cytotoxic T cells, respectively, provide crucial insights into the adaptive immune response, which is essential for understanding MASH pathogenesis [39].

**Figure 4:**
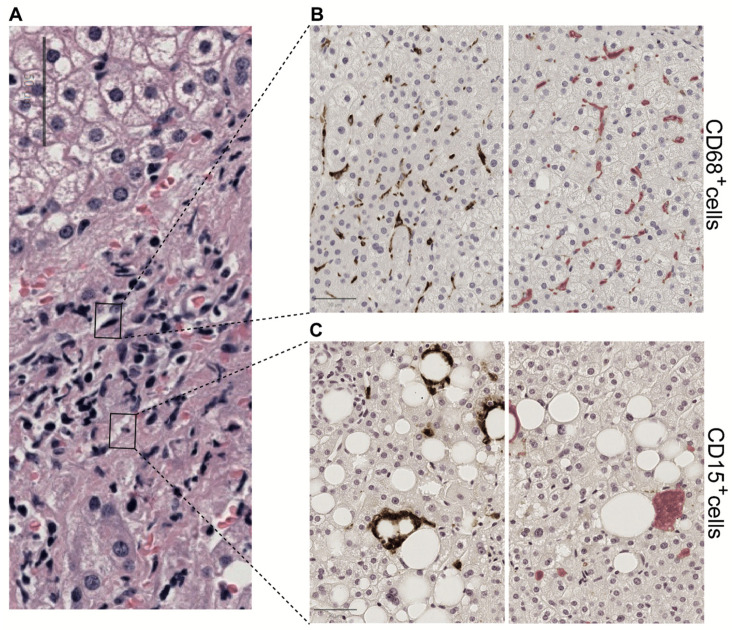
Hepatic lobular inflammation. Immunohistochemistry (IHC) techniques help characterize the inflammatory environment in the liver. Using antibodies that bind to specific antigens, IHC can highlight the presence and distribution of immune cells, identifying patterns of inflammation, cellular interactions, and the spatial organization of immune responses within the liver tissue. AI algorithms can further assist by automating the detection and quantification of specific cell populations and biomarkers, thus increasing the accuracy and efficiency of the diagnostic process. Combining IHC with advanced digital and AI techniques provides a comprehensive liver inflammation assessment. For example, in the same H&E-stained image (**A**), we notice the distribution of CD68 positive cells (**B**) and CD 15 positive cells (**C**) in tiles detected by deep learning in QuPath. This procedure facilitates the observation of circular structures surrounding areas of steatosis in perisinusoidal fibrosis. All panels are shown at 20× magnification.

By integrating these IHC markers with conventional hematoxylin and eosin staining and utilizing advanced algorithms, pathologists can compare the liver tissue’s general morphology with the spatial distribution of specific immune cells (Figure 4). This approach not only aids in diagnosis but also in monitoring the effectiveness of treatments targeting the immune response [40].

Moreover, advanced digital pathology algorithms can precisely detect color variations and apply size thresholding, enhancing assessment accuracy. Ongoing research seeks to identify the most effective markers for evaluating the innate and acquired immune responses in MASH. Differentiating between lobular and portal inflammation is another critical aspect of this evaluation, as these regions may indicate different stages or types of liver injury [41].

Machine learning algorithms offer exciting opportunities to detect, classify, and quantify cells in liver biopsies [42,43,44]. These technologies enable pathologists to annotate findings, collaborate with experts, and engage with trainees, promoting a more informed and collaborative approach to liver pathology. The integration of AI, digital pathology, and IHC represents a significant advancement in personalized patient care, particularly in the management of MASH.

### 4.4. Liver Fibrosis

Liver fibrosis can indicate future health issues and death [45]. However, there is no clear evidence of different risks between patients with and without MASH. This is important because clinical trials typically focus on patients with MASH, and improving fibrosis without worsening steatohepatitis is a primary outcome. Digital pathology may help stage liver fibrosis, but validating any method is challenging [46,47,48].

**Figure 5:**
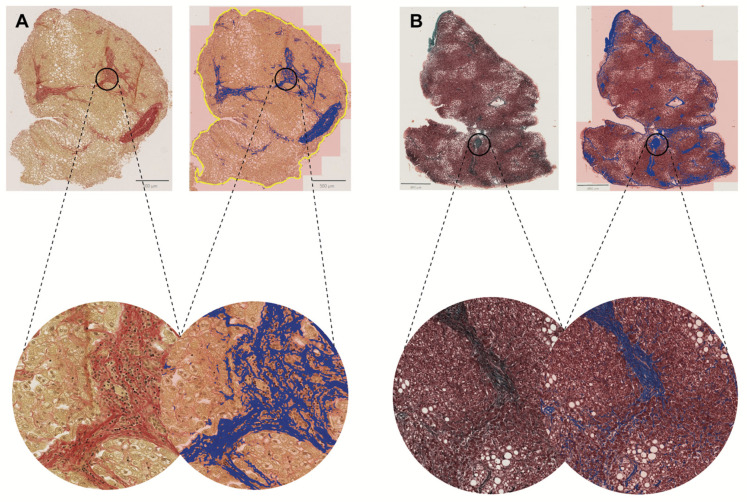
Individual fibers in fibrosis evaluation. Fibrosis can be detected using Al, which is particularly notable for its ability to identify and quantify individual fibers and their characteristics. This detailed fiber analysis is highly beneficial for continuously evaluating different stages of liver fibrosis. (**A**) Sirius Red-stained whole tissue slide at 2× magnification from a patient with F3-stage liver fibrosis. The fibrosis detection threshold created by deep learning highlights the area. (**B**) Masson’s Trichrome-stained tissue slide at 1.5× magnification from a patient with F2-stage liver fibrosis. The images in the lower part of the figure are shown at 10× magnification.

While tools are promising in quantifying fibrosis, their diagnostic value differs from traditional microscopy approaches [49,50]. There is a need for a sensitive, quantitative fibrosis analysis that identifies the total level of fibrosis and the phenotypes of fibrosis. Quantifying and calculating different collagen content, morphology, and architecture variables is important (Figure 5). Platforms like FibroNest™ offer suitable technology through a high-resolution image analysis cloud-based platform dedicated to quantifying histology-based fibrosis phenotypes. The platform can quantify 32 phenotypic traits from digitally stained slide images to establish phenotypic maps and continuous scores [51,52]. Initial findings suggest that this approach might improve the results of the preclinical efficacy studies of anti-fibrotic compounds and their translational value to clinical trials.

## 5. Unveiling the Promising Future of AI-Based Tools in Advancing Liver Research

As we increasingly integrate digital pathology into clinical practice, a trend shaping the future of liver research, this dynamic technique already addresses key aspects of experimental models of liver disease [53,54]. The use of high-resolution images reveals the intricate diversity of cells in the liver, providing insights into how hepatic cell populations proliferate, differentiate, and respond to injury [55,56,57]. This innovative approach opens new avenues in regenerative medicine and helps identify critical liver repair cells. Some researchers are even leveraging this advancement to explore the three-dimensional structure of the liver and to decode its complex vascular system [58].

Efforts to establish a multisite liver pathology informatics platform that unites medical and scientific personnel face significant challenges, highlighting the urgent need for standardization in liver pathology [59]. Obtaining high-quality images mandates precise and accurate standardization of procedures, from tissue collection to staining. This standardization is essential to minimize variability between tissue sections, which is crucial for automated measurements and serves as a foundation for consistency in liver pathology. Ensuring uniformity in color correction through control slides or algorithms is vital. Additionally, maintaining a consistent tissue thickness is equally important. Instrument and software manufacturers recognize the necessity of adopting a standard format for large image files; however, potential storage and sharing issues must also be addressed [60,61]. Ongoing research also includes denoising medical images and Self-Supervised Learning, which enables efficient prediction in healthcare [62,63].

## 6. Concluding Remarks

AI and digital pathology are poised to become essential instruments in bridging the gap between clinical research and real-world patient care. Computers offer a compelling opportunity to enhance and streamline the diagnostic process. In the assessment of MASH, lesions often overlap and vary significantly from one region to another, making it challenging to rely on a single, disease-specific grading system. This variability can hinder reproducibility among pathologists, leading to inconsistent results. Digital pathology has the potential to overcome these limitations.

Properly trained and applied AI- or ML-based tools can offer objective, consistent evaluations of therapeutic drug effects and provide highly accurate diagnoses. These technologies are not just about automating tasks but are about enhancing human capabilities, offering insights and precision that were previously unattainable. With the development of recent multi-receptor agonist therapeutics and other drugs, the importance of such diagnostic tools cannot be overstated.

As the landscape of medicine evolves, digital pathology will undoubtedly play a pivotal role in the implementation of AI-driven solutions that offer tailored, precise, and actionable insights. These innovations not only promise to revolutionize the management of MASH but also herald a new era in the treatment of a broad spectrum of liver diseases and other conditions where precision medicine is essential.

## Data Availability

The data presented in this study are available in this perspective. We are open to sharing experience, samples, and algorithms with interested research groups upon reasonable request to jorge.joven@salutsantjoan.cat.
